# Environmental Surveillance of Zoonotic *Francisella tularensis* in the Netherlands

**DOI:** 10.3389/fcimb.2018.00140

**Published:** 2018-05-08

**Authors:** Ingmar Janse, Rozemarijn Q. J. van der Plaats, Ana Maria de Roda Husman, Mark W. J. van Passel

**Affiliations:** Zoonoses and Environmental Microbiology, Centre for Infectious Diseases Control, National Institute for Public Health and the Environment, Bilthoven, Netherlands

**Keywords:** *Francisella tularensis* holarctica, tularemia, environmental surveillance, surface water, case-related sampling, genotyping, subclades, zoonosis

## Abstract

Tularemia is an emerging zoonosis caused by the Gram-negative bacterium *Francisella tularensis*, which is able to infect a range of animal species and humans. Human infections occur through contact with animals, ingestion of food, insect bites or exposure to aerosols or water, and may lead to serious disease. *F. tularensis* may persist in aquatic reservoirs. In the Netherland, no human tularemia cases were notified for over 60 years until in 2011 an endemic patient was diagnosed, followed by 17 cases in the 6 years since. The re-emergence of tularemia could be caused by changes in reservoirs or transmission routes. We performed environmental surveillance of *F. tularensis* in surface waters in the Netherlands by using two approaches. Firstly, 339 samples were obtained from routine monitoring -not related to tularemia- at 127 locations that were visited between 1 and 8 times in 2015 and 2016. Secondly, sampling efforts were performed after reported tularemia cases (n = 8) among hares or humans in the period 2013–2017. *F. tularensis* DNA was detected at 17% of randomly selected surface water locations from different parts of the country. At most of these positive locations, DNA was not detected at each time point and levels were very low, but at two locations contamination was clearly higher. From 7 out of the 8 investigated tularemia cases, *F. tularensis* DNA was detected in at least one surface water sample collected after the case. By using a protocol tailored for amplification of low amounts of environmental DNA, 10 gene targets were sequenced. Presence of *F. tularensis* subspecies holarctica was confirmed in 4 samples, and in 2 of these, clades B.12 and B.6 were identified. This study shows that for tularemia, information regarding the spatial and temporal distribution of its causative agent could be derived from environmental surveillance of surface waters. Tracking a particular strain in the environment as source of infection is feasible and could be substantiated by genotyping, which was achieved in water samples with only low levels of *F. tularemia* present. These techniques allow the establishment of a link between tularemia cases and environmental samples without the need for cultivation.

## Introduction

Tularemia is a zoonosis with a human, animal and environmental component. The disease is caused by the Gram-negative bacterium *Francisella tularensis*, which is able to infect different species of animals besides humans. Disease in humans and animals is mostly caused by subspecies *tularensis* (type A) and *holarctica* (type B) (Hestvik et al., 2015). The infectious dose is very low and infection can cause many different symptoms, ranging from fever and skin ulcers to life-threatening pneumonia. Human infections occur through direct contact with infected animals, ingestion of food or water, insect bites or exposure to aerosols or water (WHO, 2007). Disease incidence due to infections with subspecies *holarctica* has been shown to be higher near lakes and rivers (Desvars et al., 2015). *F. tularensis* has been detected in various types of surface waters and sediments (Petersen et al., 2009; Broman et al., 2011; Janse et al., 2017) where the bacterium can be hosted by free-living protozoa and may reside in biofilms (Abd et al., 2003; Sinclair et al., 2008; van Hoek, 2013).

In the Netherlands, a patient was diagnosed with tularemia in 2011, which was the first indigenous case since 1953 (Maraha et al., 2013). Since then, occasional human cases (17 in total) have been reported. Also, after surveillance of dead hares started in 2011 (Janse et al., 2017), several *Francisella*-infected hares were identified in the same period. These human and hare tularemia cases from 2011 to 2017 occurred dispersed in time and space (van de Wetering et al., 2015; Pijnacker et al., 2016; Janse et al., 2017; Zijlstra et al., 2017), which suggests a widespread occurrence and the existence of an endemic cycle of the pathogen. In 2015, environmental surveillance following a tularemia epizootic among hares in Friesland (northern Netherlands) revealed the presence of *F. tularensis* DNA in surface water and sediments (Janse et al., 2017). Surveillance data can be used to signal potential public health threats, but also to better understand the environmental components that may drive changes in the abundance of pathogenic microbes. The apparent re-emergence of tularemia could be caused by changes in the numbers or genotypes of *F. tularensis*, and by changes in transmission routes to humans or animals. In turn, such changes could be caused by changes in host populations or the environment as a result of altered land and water usage or climate. The effects of variations in reservoirs and transmission routes could be noticeable on a local scale. Biogeographical data of presence and absence may point to habitat features that could benefit growth and/or persistence of *F. tularensis*.

We performed an environmental surveillance to gain insight into the distribution of *F. tularensis* in surface waters throughout the Netherlands. Surface water samples were obtained from monitoring programs not related to disease, as well as from location-specific sampling efforts performed after reported tularemia cases among hares or humans. Presence of *F. tularensis* DNA in surface waters was investigated and genotyping was performed to confirm the presence of *F. tularensis* subspecies *holarctica* and to further identify subclades.

## Materials and methods

### Sample collection and processing

Two different environmental surveillance approaches were used to obtain three sets of surface water samples. The first approach used water samples from locations which had not been selected based on signals indicating the presence of tularemia. Based on this approach, two sets of samples were obtained. The first set (set I) of 160 samples was collected at 51 locations in 2015 by Rijkswaterstaat (RWS, the executive agency of the Ministry of Infrastructure and Water management of the Netherlands) for a research project not related to tularemia. Samples were collected from week 17 to 40 (not evenly spread) and the number of samples per location ranged between 1 and 8 (Table 1). Surface waters included mostly freshwater, but also several brackish and saltwater locations. The second set (set II) consisted of 179 surface water samples collected at 76 locations by 9 Dutch water boards in 2016. Samples were collected from week 15 to 44 (not evenly spread) and the number of samples per location ranged between 1 and 6 (Table 1). Surface waters included only freshwater and sites were chosen from the routine monitoring program for surface water quality by the cooperating water boards. Selection criteria were locations spanning diverse water types, including small water bodies, and locations where sampling was repeated in order to include temporal variation. The selection of sampling sites was unrelated to tularemia cases, although two locations in Friesland (northern Netherlands) had a geographical link with tularemia as they were situated in an area where a hare epizootic occurred in the previous year (Janse et al., 2017). However, these samples were collected almost a year after the peak of this outbreak.

**Table 1:** Surface water samples collected for environmental surveillance.

		**Samples**	**Locations**								
			**Total**	**Frequency (number of time points)**							
				**1**	**2**	**3**	**4**	**5**	**6**	**7**	**8**
I (2015)		160	51	11	9	13	10		5	2	1
II (2016)		179	76	19	29	24			5		
III (2013-17)	MB (2013)	4	4	4							
	FL (2015)	77	42	26	4	8	2	2			
	MV (2015)	5	4	3	1						
	ZL (2015)	5	5	5							
	HB (2016)	14	14	14							
	FP (2016)	8	7	6	1						
	LB (2016)	5	5	5							
	RH (2017)	9	9	9							

*The table displays for each dataset (I, II and III) the total number of samples, the total number of locations where these were collected and the number of locations where sampling was repeated between 1 to 8 time points*.

In the second environmental surveillance approach, sampling was performed near locations of notified tularemia cases in humans or hares. Based on this approach, a third set of 130 samples (set III) was collected in 8 different geographical regions in the period from 2013 to 2017 (Table 1). Locations for casus-related sampling were selected as follows. In the Netherlands, tularemia is a notifiable disease in humans since November 2016, but in the preceding period, cases were also monitored (Janse et al., 2017). In the period from 2015 to 2017, there were several human tularemia cases with a potential environmental infection source. In the same period, dead hares were also investigated for tularemia, which resulted in the recognition of several confirmed tularemia cases. Eight of these human or hare signals which could be linked to a possible exposure site were followed up by the collection of between 4 and 77 surface water samples (Table 1). Indications for sampling locations based on human cases ranged from the home address of a patient who had not had direct contact with water to an obvious exposure during a mud run event (Zijlstra et al., 2017). Sampling locations based on tularemia confirmed in dead hares was based on the finding location of these hares. After 5 cases, samples were collected at one single time point, whereas after 3 cases follow-up sampling was carried out as well (Table 1). A follow-up sample collected at Maarsseveen (MV) was included because a second human tularemia case possibly linked to environmental exposure had been recognized in the same area. In Friesland (FL) and in the Flevopolder (FP), follow-up samples were collected because of the relatively high levels of *F. tularensis* DNA in the first surface water samples. Most results from the Friesland cluster were described in an earlier communication (Janse et al., 2017), but the current report includes additional samples from a wider area.

Water samples collected in 1 L flasks were transported to the laboratory. Water was filtered using Tuffryn membrane filters (Pall Life Sciences, Ann Arbor, USA) with a pore size of 0.45 μm until they clogged, after which the filters were stored at −20°. DNA was extracted from the filters by using the PowerWater DNA extraction kit (Qiagen, Hilden, Germany) according to the manufacturer's recommendations.

### Detection of *F. tularensis* DNA

To detect *F. tularensis* DNA, both undiluted and 10x diluted extracts were analyzed in triplicate by using qPCR (Janse et al., 2010). This multiplex assay includes multi-copy signature sequence IS*Ftu*2 and single-copy gene *fop*A for the detection of *F. tularensis* species. Subspecies *holarctica, tularemia, novicida* and *mediasiatica* are all detected. Marker (*pdp*D) for differentiation between subtypes A (*F. tularensis tularensis*) and B (*F. tularensis holarctica*) was not included in the assay because subtype A is not encountered in Europe and it was not detected in surface water samples analyzed previously (Janse et al., 2017). To enable comparison of DNA levels between samples that can also be easily visualized, positive qPCR results were binned into 5 categories as follows. Level 1 = IS*Ftu*2 detected 1 or 2 out of 3; Level 2 = IS*Ftu*2 detected 3 out of 3; Level 3 = IS*Ftu*2 detected 3 out of 3 and Cq value < 33; Level 4 = IS*Ftu*2 detected 3 out of 3 and *fop*A detected 1 or 2 out of 3; Level 5 = Both IS*Ftu*2 and *fop*A detected 3 out of 3. Binning was based on the fact that increasing amounts of *F. tularensis* gDNA subsequently increase the chance of detecting target in each replicate reaction, result in lower Cq values and increase detection of single-copy target *fop*A in addition to multicopy target IS*Ftu*2.

### Sequence analysis

Primers were used for amplification of *F. tularensis* DNA for subsequent sequence analysis. Sequences that were targeted included the genes used for the detection (*ISFtu2* and *fop*A), and a selection of 8 additional genes which could be used for the differentiation of subclades. Gene selection was based on studies developing genotyping assays (Svensson et al., 2009; Vogler et al., 2009; Birdsell et al., 2014). Primers for the amplification of *ISFtu2* and *fop*A had been described previously (Janse et al., 2010) and spanned larger gene fragments (524 and 428 bp, respectively) than those used for qPCR detection (89 and 115 bp, respectively). Novel oligonucleotides were designed using the software package Visual Oligonucleotide Modeling Platform (DNA software Inc. Ann Arbor, USA) for application in a multiplex mixture of 20 primers and amplifying a region of between 250 and 350 basepairs. Gene targets, primer sequences and amplicon sizes are displayed in Supplemental Table S1.

Sequences were amplified by using a two-step protocol. In the first step, amplification was performed by using the SSO pre-amplification kit (Bio-Rad, California, USA) in reactions containing all 20 primers mixed at a final concentration of 50 nM each. Thermocycling conditions were as follows: 95°C for 3 min, 14 cycles at 95°C for 15 s, 56°C for 240 s. Thermocycling reactions were carried out in a C1000 Touch combined with a S1000 Thermal Cycler (Bio-Rad, California, USA). Subsequently, primers were removed by incubation with 0.6 U/μl final concentration Exonuclease I (New England Biolabs, Massachusetts, USA) 37°C for 30 min, followed by enzyme inactivation at 80°C for 15 min. The reaction was diluted 5x in Tris-EDTA buffer solution and these amplified materials were used for a second step during which each target sequence was amplified in a separate PCR reaction. The Qiagen multiplex PCRkit (Qiagen, Hilden, Germany) was used for this amplification, primers were present at a final concentration of 200 nM. Thermocycling conditions were as follows: 95°C for 15 min, 35 cycles at 94°C for 30 s, 57°C for 90 s, and 72°C for 90 s, followed by a final step at 72°C for 10 min. Quality and quantity of PCR products were inspected on the Agilent 2100 Bioanalyzer instrument using the DNA 1000 kit (Agilent Technologies, Eindhoven, the Netherlands). To prevent cross-contamination between samples, rigorous PCR protocols were applied. Moreover, materials from control strains were utilized at low concentrations and control materials and environmental samples were processed in separate experiments. PCR products were purified by using ExoSAP-IT (USB, Cleveland, USA) and Sanger sequencing of both strands was performed by Baseclear (Leiden, the Netherlands). Sequences were deposited in Genbank under accession numbers MH156230-MH156254. Strand assembly, identification of genomes containing similar sequences by using BLAST, genome retrieval from NCBI and sequence alignment was carried out using CLCbio software (Qiagen, Hilden, Germany). The CanSNPer program (Lärkeryd et al., 2014) was used for nomenclature of the canonical SNPs with strain OSU18 (accession CP000437) as reference genome. As a positive control for amplification and sequencing of gene targets, we used strains LVS, *F. tularensis holarctica* clade B.12 (subclade B.23) and Ft7, *F. tularensis holarctica* clade B.6, which has been isolated from a Spanish patient.

## Results

**Figure 1:**
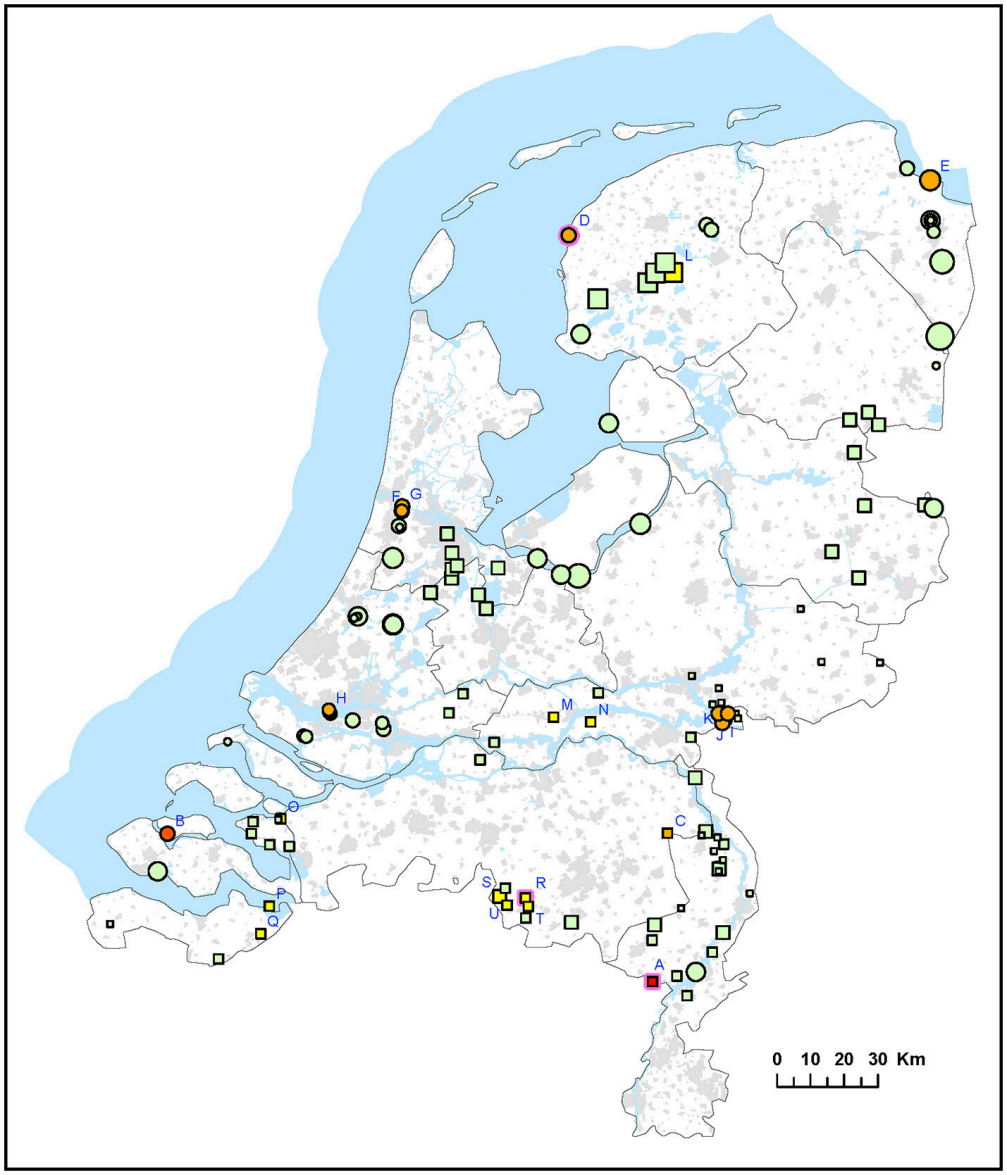
Occurrence of *F. tularensis* in surface waters in the Netherlands. Circles represent samples collected in 2015 (sample set I) and squares represent samples collected in 2016 (sample set II). Symbol sizes correlate to the number of repeated samples obtained from a particular location (range 1-8). Colors refer to the level of *F. tularenis* DNA in the samples and ranges from 0 (DNA not detected) to 5 (highest level). Green = 0, yellow = 1, light orange = 2, dark orange = 3, light red = 4, bright red = 5. The level was based on the detection of multicopy target IS*Ftu*2 and singlecopy target *fop*A in triplicate qPCR measurements. When sampling was repeated, symbol color was based on the time-point with the highest DNA level. A pink halo indicates that *F. tularemia* DNA was detected at each time-point. Blue capitals cross-reference to Table 2 which gives more details on the water samples in which *F. tularensis* was detected.

Two different approaches were used for environmental surveillance for *F. tularensis* in the Netherlands. For the first approach, sampling locations were selected independent of recent tularemia cases. A total of 339 surface water samples were collected from 51 locations in 2015 (set I) and from 76 locations in 2016 (set II). The frequency of sampling at each location varied between 1 and 8 (Table 1). *F. tularensis* DNA was detected by using qPCR and different levels of contamination were recognized by binning positive results into 5 categories of increasing DNA concentrations (Figure 1 and Table 2). *F. tularensis* DNA was detected at 22 out of the 127 randomly selected surface water locations (17%) in different parts of the country. Positive locations included three brackish or saltwater locations (Figure 1, D, E, P). The level detected was highest at two locations (Figure 1 and Table 2, A and B), with at location A detection of the single-copy gene target *fop*A in addition to the detection of multi-copy signature sequence IS*Ftu*2 (Janse et al., 2010). At three locations which were sampled more than once (i.e., all but the smallest symbols in Figure 1), *F. tularensis* DNA was detected at all 3 (location D) or 2 (location A and R) time-points (Table 2 and Figure 1). In contrast, most of the positive locations that were sampled repeatedly included at least one time point when *F. tularensis* was not detected. This was true when samples had been collected at 6 (locations E and L), 3 (locations B, G, I, J, K, S) or 2 (locations C, F, H, M, N, O, P, Q, T, U) time-points (Figure 1 and Table 2). There was no significant correlation between the time of the year samples were collected and detection of *F. tularensis* DNA (data not shown).

**Table 2:** Details (locations and timepoints) of *F. tularensis* DNA detection in surface water samples collected in 2015 (set I) and 2016 (set II).

**Sample information**						**April**			**May**					**June**				**July**				**August**				**September**				**October**			
**Code Figure 1**	**MaxScore**	**Set**	**Surface water type**	**year**	**week**	**15**	**16**	**17**	**18**	**19**	**20**	**21**	**22**	**23**	**24**	**25**	**26**	**27**	**28**	**29**	**31**	**32**	**33**	**34**	**35**	**36**	**37**	**38**	**39**	**40**	**41**	**42**	**43**
A	5	II	Canal	2016																		5					4						
B	3	I	Lake	2015																3			2				0						
C	2	II	Small canal	2016																		2				0							
D	2	I	Estuary	2015																1	2		2										
E	2	I	Estuary	2015												1		0		1	2		0				2						
F	2	I	Small lake	2015												0		2															
G	2	I	Small lake	2015																2	0		1										
H	2	I	River	2015											0				2														
I	2	I	Lake	2015																				0		0		2					
J	2	I	Lake	2015																				1		0		2					
K	2	I	Lake	2015																				2		0		2					
L	1	II	Ditch	2016				1				0				0				0			0				0						
M	1	II	Small lake	2016																								1		0			
N	1	II	Ditch	2016																									0				1
O	1	II	Canal	2016			0					1																					
P	1	II	Estuary	2016			0							1																			
Q	1	II	Small canal	2016		0					1																						
R	1	II	Canal	2016																			1				1						
S	1	II	Small lake	2016																			1		0		0						
T	1	II	Canal	2016																			0				1						
U	1	II	Canal	2016																		0					1						
V	1	II	Canal	2016										1																			

*Numbers refer to the levels of F. tularenis DNA detected in the samples, ranging from 0 (not detected) to 5 (highest level)*.

**Figure 2:**
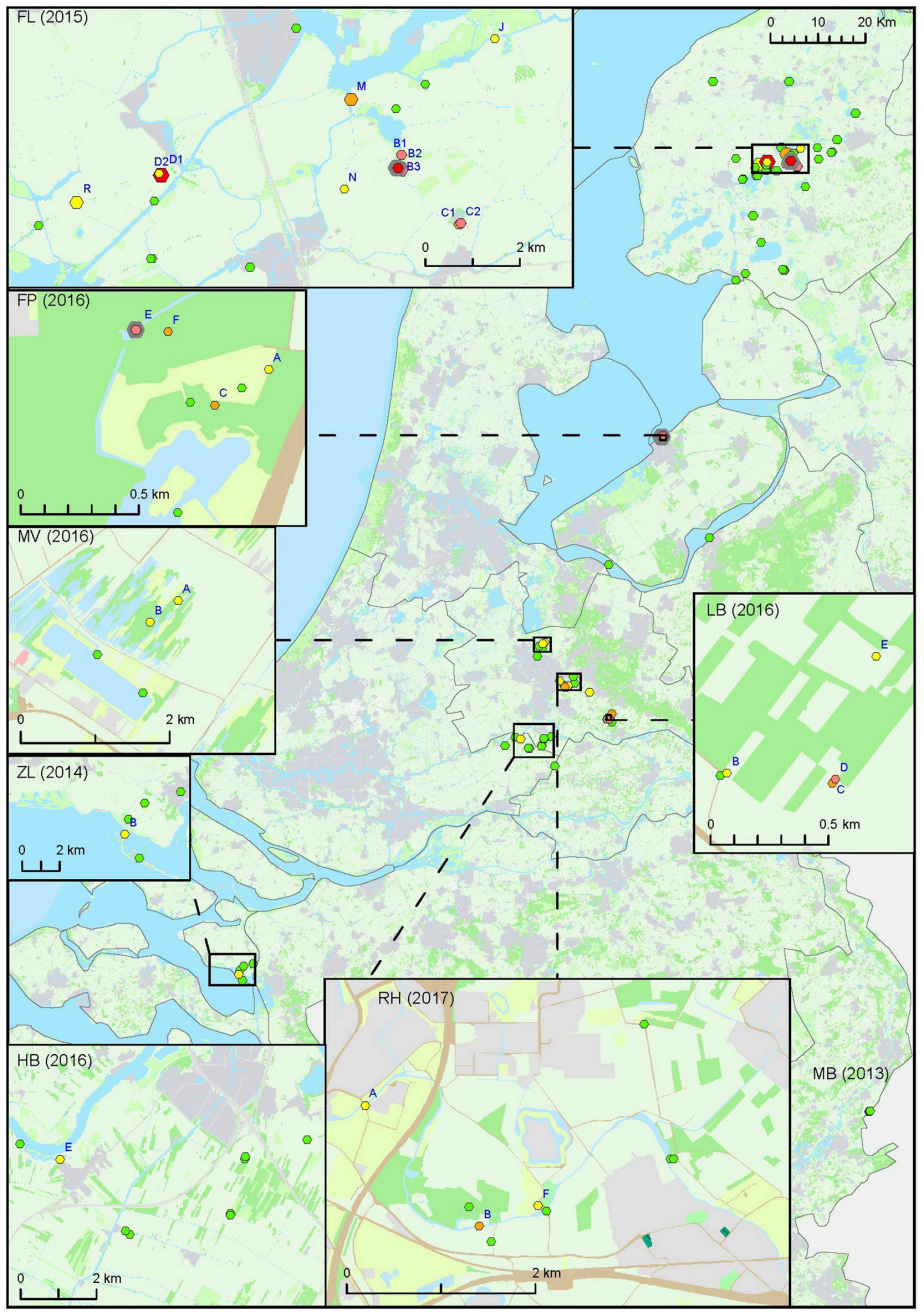
Occurrence of *F. tularensis* in surface waters in the Netherlands following tularemia cases in humans or hares. Samples were collected between 2013 and 2017 (sample set III). Symbol sizes correlate to the number of repeated samples obtained from a particular location (range 1-8). Colors refer to the level of *F. tularensis* DNA in the samples and ranges from 0 (DNA not detected) to 5 (highest level). Green = 0, yellow = 1, light orange = 2, dark orange = 3, light red = 4, bright red = 5. The level was based on the detection of multicopy target IS*Ftu*2 and singlecopy target *fop*A in triplicate qPCR measurements. When sampling was repeated, symbol color was based on the time-point with the highest DNA level. A pink halo indicates that *F. tularensis* DNA was detected at each time-point. Blue capitals cross-reference to Table 3 which gives more details on tularemia cases and water samples in which *F. tularensis* was detected.

For the second approach, a total of 127 surface water samples were collected following reported tularemia cases in hares or humans with a possible environmental source of the infection. In 7 out of 8 of such tularemia case-related sampling efforts, *F. tularensis* DNA was detected at one or more surface water locations (Figure 2 and Tables 1, 3). *F. tularensis* DNA was detected even though the period between suspected infection and sample collection could be up to 6 weeks (Table 3). *F. tularensis* DNA was not detected in samples collected more than 2 months after a case in the South-East of the Netherlands in 2013 (MB; Table 1, Figure 2). On the other hand, it was detected in the South-West, where samples were collected one year after tularemia cases were reported from the area (ZL; Tables 1, 3, Figure 2). Higher DNA levels, including levels permitting the detection of single-copy gene *fop*A, were found after 3 tularemia cases: FL, FP and LB (Table 3 and Figure 2).

**Table 3:** *F. tularensis* DNA in surface water samples collected at locations with a history of tularemia cases.

**case**				**surface water samples**											
**Infected host**	**Insert Figure 2**	**Year**	**Week**	**Year**	**Code sample**	**Week**	**Ft**	**Week**	**Ft**	**Week**	**Ft**	**Week**	**Ft**	**Week**	**Ft**
hare	FL^a^	2015	7–20^b^	2015	FL-B1	16	4	22	0						
					FL-B2	16	4	22	2	31	0	34	0	37	0
					FL-B3	16	5	22	4						
					FL-C1	16	2	22	0						
					FL-C2	16	4	22	0						
					FL-D1	16	5	22	2	31	1	34	0	37	0
					FL-J			22	1						
					FL-M			22	2	31	0	34	0	37	0
					FL-N			22	1						
					FL-R			22	1	31	0	34	0	37	0
human	FP^a^	2016	35	2016	FP-A	40	1								
					FP-C	40	2								
					FP-E	40	4	44	4						
					FP-F	40	2								
human	MV	2015	33	2015	MV-A	39	1								
					MV-B	39	1								
hare	LB	2016	3, 41	2016	LB-B	44	1								
human			10		LB-C	44	2								
					LB-D	44	4								
					LB-E	44	1								
hare	HB	2014	15	2016	HB-M	25	2								
human		2016	19		HB-N	25	1								
					HB-E	25	1								
hare	RH	2017	20	2017	RH-A	25	1								
					RH-B	25	2								
					RH-F	25	1								
hare	ZL	2014	9–14^c^	2015	ZL-B	22	1								
human			9–14^c^												

*Numbers refer to the level of F. tularenis DNA detected in the samples, ranging from 0 (not detected) to 5 (highest level)*.

a *Case references; FL: (Janse et al., 2017), FP: (Zijlstra et al., 2017)*.

b *Multiple hares were found during this period*.

c *Only month in which case occurred is known*.

Sample collection at most locations was not repeated, with two exceptions. During the hare epizootic in Friesland mentioned above, initial sampling at several locations was followed by a second sample collection after 1 month, and locations both from within the epizootic area and from more distant locations were sampled at monthly intervals for 3 months after the epizootic had ceased. Also, after detection of *F. tularensis* DNA in surface water linked to a tularemia case with suspected environmental exposure, a follow-up sample was collected 1 month later (Table 3, FL and FP). At those locations with relatively high *F. tularensis* levels detected in the initial samples (Table 3, FL-B2, FL-B3, FL-D1 and FP-E), *F. tularensis* DNA was again detected in the follow-up sample. However, after the hare epizootic in Friesland ended, *F. tularensis* was no longer detected (Figure 1 and Tables 2, 3).

In order to confirm the presence of *F. tularensis* subspecies *holarctica*, and to enable typing to subclade level (Svensson et al., 2009; Vogler et al., 2009), sequences were obtained from DNA amplified from selected surface water samples. These samples included sample A from set II (Figure 1 and Table 2) and FL-B3, FL-D1, FP-E, LB-D from set III (Figure 2 and Table 3). Ten DNA targets (Supplemental Table S1) were sequenced from samples A and FL-D1, whereas from samples FL-B3, FP-E, and LB-D only target sequence IS*Ftu*2 was sequenced.

Sequence similarities between gene targets amplified from water samples and from two reference strains (Ft7 and LVS) were investigated and compared to reference sequences. Sequences from gene target IS*Ftu*2 amplified from samples FL-D1, FL-B3, and LB-D were identical and matched those of *F. tularensis holarctica* strains while they had mismatches with IS*Ftu*2 sequences from other *F. tularensis* substrains. In contrast, IS*Ftu*2 sequences amplified from samples A and FP-E were very different from the other samples (13 and 11% mismatches, respectively). BLAST analysis showed for samples A and FP-E highest similarity with genomes from *F. hispaniensis* (Huber et al., 2010) (6.1 and 4.7%, respectively) and *Francisella uliginis* sp. nov. (Challacombe et al., 2017) (6.7% and 5.9% mismatches, respectively). Sequences from gene target *fop*A amplified from samples FL-B3, FL-D1 and A were identical and matched those of *F. tularensis holarctica* and *F. tularensis novicida* strains. All sequences from gene targets *put*A, FTH_0021, *pdp*C1, *rib*A, FTH_0165, FTH_1370, *aro*A, and *gph*-*lys*R (Supplemental Table S1) amplified from samples FL-D1 and A matched with *F. tularensis holarctica* strains. Several sequence positions had mismatches with all other *F. tularensis* species and subspecies and were thereby exclusive for *F. tularensis holarctica*. Sequence variation between *F. tularensis holarctica* strains at particular positions was used to identify subclades (Svensson et al., 2009; Lärkeryd et al., 2014). In sample FL-D1, the derived base G in target pdpC1 (position 107819 in strain OSU18, accession CP000437) points to the presence of *F. tularensis holarctica* clade B.12 (subclade B.20). Similarly, in sample A, *F. tularensis holarctica* could be classified as clade B.6(indel Ftind49) based on a 9 bp deletion TGGCAATTT (position 1339960–68 in strain OSU18, accession CP000437).

## Discussion

### Spatial and temporal variation

*F. tularensis* appeared to be present at various locations throughout the Netherlands, including freshwater, brackish water and saltwater. Locations could be identified where occurrence was more prominent, as evidenced from higher levels of *F. tularensis* DNA detected and recurring detection when sampling was repeated. Most of these locations were derived from case-related surveillance (Figure 2 and Table 3). Case-unrelated surveillance yielded only two such locations with relatively high *F. tularensis* contamination (A and B, Figure 1, Table 2).

It is not possible to conclude absence of *F. tularensis* at a particular location. This is illustrated by the fluctuation of presence and absence between subsequent time points, which was often observed at locations with low levels of *F. tularensis* (Tables 2, 3). Nevertheless, at several locations a presence of *F. tularensis* could be considered less likely as its DNA was not detected, including after repeated sampling. No common habitat features were identified for locations with different levels of *F. tularensis* contamination. Previous research had suggested a more likely *F. tularensis* occurrence in smaller water bodies such as ditches, since *F. tularensis* DNA was not detected in samples from lakes and larger canals (Janse et al., 2017). However, since the selection of sampling locations for that study was largely based on finding sites of tularemia confirmed dead hares, smaller water bodies in rural areas were more frequently investigated. The selection of case related sampling locations (set III) in the current study was subject to a similar bias. Although 5 sampling locations were also based on human cases, the two tularemia patients with an obvious surface water exposure (swimming and a mud run) had had contact with small water bodies. The different approach used for the selection of locations for sample sets I and II in this study yielded a more diverse array of surface waters. Results from these 127 locations showed that *F. tularensis* DNA can be detected in large water bodies, including at the banks of lakes (B, Figure 1) and at sea shores (D, E, Figure 1).

The occurrence of *F. tularensis* in diverse aquatic environments, possibly involving biofilms, has been reported before (Sinclair et al., 2008; Petersen et al., 2009; Broman et al., 2011; van Hoek, 2013). Its widespread occurrence suggests diverse roles in aquatic ecosystems, which may also differ between strains and relate to their ability to associate with unicellular eukaryotes (Duodu et al., 2012). In addition, at least some of the distribution of *F. tularensis* may be explained by transient contamination by shedding from infected animals. Animals with a high bacterial load may act as amplifiers contaminating the local environment (Broman et al., 2011; Rossow et al., 2014; Schulze et al., 2016). Such contamination will be difficult to verify as tularemia in wild animals will largely go unnoticed. For instance, in the Netherlands, only hares that died from tularemia have a slight chance of being noted.

Besides surface waters, infected animals such as rodents could also contaminate local (open) water supplies. Therefore, small, uncontrolled private supplies could be potential exposure routes for tularemia. In Sweden and Turkey, consumption of untreated drinking water from private wells and small community supplies has been identified as source of infection (Karadenizli et al., 2015; Lindhusen Lindhé et al., 2018).

### Environmental surveillance

Presence of *F. tularensis* in surface water may imply health risks. Tularemia incidence was shown to correlate with the presence of aquatic habitats (Desvars et al., 2015). Also, the occurrence of *F. tularensis* in surface water and sediment has been associated with human tularemia outbreaks (Broman et al., 2011). However, it is not possible to infer or predict infection risk or tularemia incidence based on our results of *F. tularensis* DNA levels in surface water. This is due to several factors, such as a limited number of cases and samples, variable time-periods between cases and sampling and absence of information about viability and infectivity of *F. tularensis* detected by using qPCR. Nevertheless, data from the environmental surveillance approach independent of tularemia cases (Figure 1 and Table 2) could be used to identify locations harboring higher levels of *F. tularensis*, which could be useful to focus research of tularemia incidence in relation to occurrence of *F. tularensis*.

Our data support the feasibility of tracking possible sources of environmental exposure. It was more likely to detect *F. tularensis* in surface water samples obtained following a tularemia case (set III) compared with case-unrelated samples (set I and II). Positive samples were obtained from 7 out of 8 (88%) case-related sampling efforts, while from randomly collected samples only 17% of the locations (and 10% of the samples) were positive. These figures can only be used to illustrate these differences, as the datasets differ too much in numbers of samples (Table 1) and definition of locations (Figures 1, 2) to support quantitative comparisons.

A detailed environmental investigation following a reported case could point to the most likely source of infection on a local scale. For instance, several locations that were investigated in relation to the FP case clearly showed different levels of *F. tularensis* contamination (Figure 2 and Table 3). Findings from the outbreak among hares in Friesland also showed that detection of *F. tularensis* following tularemia cases is limited to a geographical area and time period (Figure 2 and Table 3) (Janse et al., 2017). This is also congruent with a study in Germany where *F. tularensis* DNA was detected in surface water samples after animal tularemia cases, but not at distant sites or in the following year (Schulze et al., 2016). These findings support the identification of a particular strain as the source of infection if retrieved from surface water samples collected after tularemia cases. A link between source and case could be substantiated by genotyping, which can be done using cultivated isolates (Karadenizli et al., 2015), but if these are not available also by directly analyzing water samples (see below).

### Genotyping *F. tularensis* holarctica in surface waters

Presence of *F. tularensis holarctica* in samples FL-D1, FL-B3 and LB-D was confirmed by the amplified IS*Ftu*2 sequences, which differ at several positions from other subspecies. One useful signature is a deletion TAG which corresponds to *Francisella tularenis tularenis* strain SCHU S4 (accession AJ749949) position 103552–103554. In contrast, IS*Ftu*2 sequences from samples FP-E and A had a low similarity to those from *F. tularensis holarctica* and were likely amplified from an unknown and abundant environmental strain. Inspection of primer binding sites in the most similar genome from *Francisella hispaniensis* strain FSC454 (accession CP018093), revealed only 1 mismatch with both the forward and reverse primers used for IS*Ftu*2 amplification. Therefore, it is likely that DNA from a similar strain present in higher numbers than *F. tularensis holarctica* was amplified preferentially, thereby preventing amplification of IS*Ftu*2 from *F. tularensis holarctica*. This illustrates that it is important to be aware of the primer matches with (hitherto unknown) related non-target organism (Ahlinder et al., 2012; Challacombe et al., 2017). For future studies encompassing sequencing of IS*Ftu*2 from *F. tularensis holarctica* in environmental samples, it is advisable to adjust sequencing primers to make them more selective. Inspection of the primers used for detection of *F. tularensis* confirmed that the specificity of the qPCR detection of *F. tularensis* was not compromised by the presence of this environmental strain, as the reverse primer and probe had respectively 7 and 4 mismatches with these sequences (data not shown). Therefore, the *Francisella spp*. of indirect relevance to tularemia ecology and epidemiology that were detected in two of the samples did not impact the qPCR detection of *F. tularensis* in our environmental surveillance. The other gene targets from which sequences were obtained, i.e., all targets for samples FL-D (set III) and A (set I) and targets *fop*A and FTH_0165 for sample FL-B3 (set III), all confirmed the presence of *F. tularensis holarctica* in the water samples. Sequences were identical to sequences derived from *F. tularensis holarctica* genomes, and there were several sequence positions which differed from all other *Francisella* strains. In summary, sequencing confirmed the presence of *F. tularensis holarctica* in 4 water samples that were investigated in more detail. These samples were derived both from case-related and unrelated surveillance efforts. Presence of *F. tularensis holarctica* was not confirmed in sample FP-E, but this was probably due to the fact that only target IS*Ftu*2 was inspected for this sample. More extensive sequencing efforts, as was carried out for sample A, would likely identify *F. tularensis holarctica*.

Investigation of all gene targets as was carried out with samples FL-D1 and A, permitted differentiation between locations by typing to the subclade level (respectively clade B.12, SNP B.20 and clade B.6, indel Ftind49). The identification of *F. tularensis holarctica* clade B.6 supports the possibility of surface water as source of infection of the first endemic case from 2011, since a clinical isolate obtained from this patient was classified as this Franco-Iberian subclone (Maraha et al., 2013).

Because the methods used only reveal dominant sequences, the presence of other subclades in a sample cannot be ruled out. Considerable genomic diversity of *F. tularensis* has been shown in e.g., Scandinavian countries and Germany (Karlsson et al., 2013; Schulze et al., 2016), and similar diversity may occur in the Netherlands. A more detailed investigation of *F. tularensis* diversity, including less abundant genotypes, would require different protocols however, including clonal purification of DNA targets and NGS sequencing methods. We showed that it is possible to perform such genotyping in samples in which only low levels of *F. tularensis* were detected. This prevents the need for isolation of strains from the environment, which can be very difficult because of low absolute and relative numbers of *F. tularensis* and abundant competing bacteria able to grow in isolation media. By applying these techniques to tissue samples or isolated strains derived from patients or animals, it will be possible to establish a link between tularemia cases and environmental samples.

## Conclusions

This study shows that for tularemia, valuable information regarding the spatial and temporal distribution of its causative agent could be derived from environmental surveillance. The significance of detectable levels of *F. tularensis* in surface waters in terms of infection risks requires more immediate and extensive monitoring data to relate to information about tularemia cases. Tracking a particular strain as source of infection from an environmental source is feasible and could be substantiated by genotyping, which was shown to be possible directly on water samples with only low levels of *F. tularensis* present.

## Author contributions

IJ designed the study, carried out sampling, analyzed data and wrote the manuscript. RP performed the experiments and contributed to the design of the study. AR provided intellectual input. MP contributed to design of the study and intellectual input. All authors contributed to manuscript revision, read and approved the submitted version.

### Conflict of interest statement

The authors declare that the research was conducted in the absence of any commercial or financial relationships that could be construed as a potential conflict of interest.
