# Supplementary material for: Environmental Surveillance of Zoonotic Francisella tularensis in the Netherlands
Source: Front Cell Infect Microbiol. 2018 May 8;8:140. doi: 10.3389/fcimb.2018.00140 (PMC5951967; doi:10.3389/fcimb.2018.00140)
Supplement: Supplementary file 1 [file Table_1.DOCX]

Supplemental table S1. Gene targets and primer sequences used for amplification and sequencing of *F. tularensis*.

| **OSU18 locus ID^a^** | **OSU18 gene^a^** | **Clade^b^** | **SNP/**  **indel^b^** | **forward primer sequence (5'-3')** | **reverse primer sequence (5'-3')** | **amplicon size** |
| --- | --- | --- | --- | --- | --- | --- |
| FTH_0042…FTH_1847 (38 loci) | IS*Ftu*2 |  |  | ATGGTAAATATAGATCAATACATAAGCGTT | GGTTTTATCAAATCTAGAGAATACTCTTC | 524 |
| FTH_1293 | *fop*A |  |  | GATGAGATTAAAAAGTATTGTTATAGCTAC | CTGCAGCATATGGAGTAAACATAGTAT | 428 |
| FTH_0799 | *put*A |  | T.1 | CATACTCGATCATAAACGCA | TTAAAGTAGCTAAAAGGTGTGT | 257 |
| FTH_0072 | *rib*A | **B.4** | B.17 | GAAATGATTAGCGAAGAGGG | GTGTTTCTATATCAACGTTTCT | 271 |
| FTH_1370 |  | **B.6** | Ftind49 | GCAGGCTTTGAATCACTTGA | TTCTAGTATCATCAACGTCCC | 331 |
| FTH_0165 |  | **B.12** | B.23 | GTGAGTTCCAACAAATAAATACCAC | GAAGATCCTTTAGAAGTTCATCGAC | 300 |
| FTH_1134  FTH_0108 | *pdp*C1  *pdp*C2 | **B.12** | B.20 | TGGGTCGGACTATCACATCAA | TTCTATTACTGGTTTTGAGGC | 267 |
| FTH_1517  FTH_1516 | *gph*-*lys*R | **B.12** | Ftind33 | GCCAAAACATCTTCAGATACAGGA | TATCCGTCCGATCAGTTTCAACC | 264 |
| FTH_0021 |  | **B.16** | B.2 | TAATTCAAGGTTTTTCATTCGG | GTTTCTTGAATTGAAGCACG | 265 |
| FTH_0841 | *aro*A | **B.16** | B.16 | TGAGATTTGTTGGCAGTATAGA | AAGCAAAGAATGATGGATAGAC | 332 |

^a^ F. tularensis tularensis strain OSU18, accession CP000437.

^b^ Table 2 and 3, Figure 3 in: Svensson, K. et al. 2009. A real-time PCR array for hierarchical identification of *Francisella* isolates. PLoS One 4(12).
